# The Late Emperor of Germany

**Published:** 1888-07

**Authors:** 


### THE LATE EMPEROR OF GERMANY.

The question as to the nature of the disease which has caused the death of the late emperor of Germany is, if we may judge by the brief notes received by the secular press by cable, answered by the autopsy.

This statement of the post-mortem examination substantiates the opinion which has been held by the greater portion of those members of the profession best qualified to judge of the case. The course, and in general the history, of the case does not materially differ from that of most cases of cancer of the larynx. The interest of this particular case consists in the fact that the patient was the heir to the throne of one of the greatest empires of Europe.

There will always in this instance, as there must in all other similar cases, remain a doubt as to one point, namely, the bearing of the treatment upon the duration of life. The early recognition of the nature of the disease could in no way solve that question. In too many cases an operation has been followed by a speedy dissolution. The average duration of life in general has been quite as long, or perhaps we may say longer without any operation except that of tracheotomy, as when there has been made an effort to extirpate the larynx, or even a portion of it. On the other hand the effort to remove the larynx, or the diseased portion of it, has evidently in many instances shortened life. Whether in this case such would have been the result, it is, of course, impossible to know. All things considered it is quite certain that the management by Mackenzie and his aids materially prolonged the life of the illustrious sufferer and secured for him the maximum of comfort and ability to continue to the very last in the discharge of the functions of his high estate.

The position of the German profession toward the eminent English surgeon is a little uncertain. The secular press has represented this attitude as one of hostility, of bitter criticism. The German journals which we have been able to consult hardly justify such a statement. The most eminent of the profession in Germany have not been quite certain as to the nature of the trouble. The examination of the portions removed by Mackenzie by the master of pathology not only among the Germans themselves, but one recognized by the whole learned world as competent, if any one can be, to decide the case, left it in doubt whether the growth was a cancer or a non-malignant tumor. Of course, the failure to find evidence of malignancy did not in the mind of any one exclude the possibility of malignancy, but this fact raised the hope that the condition might not be cancer, and justified treatment directed to the amelioration of the symptoms and calculated to prolong life.

The charge has been made that Mackenzie recognized at once the character of the disease, but gave a false diagnosis and prognosis for political reasons and under the influence of the princess, the wife of the patient. No one who has had the privilege of knowing the celebrated specialist who has been placed in this trying position will for a moment believe that he in any way departed from the line of moral or professional obligation, even to serve a throne. The charge that he had misstated the facts does not seem to be borne out even by the admitted history of the case. He simply recognized the truth that there was a doubt as to the nature of the malady, and hoped that the case was not malignant. Had the operation been performed, as contemplated by the German attendants when Mackenzie was called, no one, of course, can say that the emperor would not now be living, but it is much more probable that he would have been dead months before, and that he would never have come to the throne.

